# Reduction of Relapse after Unrelated Donor Stem Cell Transplantation by KIR-Based Graft Selection

**DOI:** 10.3389/fimmu.2017.00041

**Published:** 2017-02-08

**Authors:** Silke Heidenreich, Nicolaus Kröger

**Affiliations:** ^1^Department of Stem Cell Transplantation, University Medical Center Hamburg-Eppendorf, Hamburg, Germany

**Keywords:** NK-cell, killer-cell immunoglobulin-like receptor, unrelated, stem cell, transplantation, HSCT, haplotype

## Abstract

Besides donor T cells, natural killer (NK) cells are considered to have a major role in preventing relapse after allogeneic hematopoietic stem cell transplantation (HSCT). After T-cell-depleted haploidentical HSCT, a strong NK alloreactivity has been described. These effects have been attributed to killer-cell immunoglobulin-like receptors (KIR). Abundant reports suggest a major role of KIR not only on outcome after haploidentical HSCT but also in the unrelated donor setting. In this review, we give a brief overview of the mechanism of NK cell activation, nomenclature of KIR haplotypes, human leukocyte antigen (HLA) groups, and distinct models for prediction of NK cell alloreactivity. It can be concluded that KIR-ligand mismatch seems to provoke adverse effects in unrelated donor HSCT with reduced overall survival and increased risk for high-grade acute graft-versus-host disease. The presence of activating KIR, as seen in KIR haplotype B, as well as the patient’s HLA C1/x haplotype might reduce relapse in myeloid malignancies.

## Introduction

Natural killer (NK) cells are considered to contribute important immune effects against leukemia [graft-versus-leukemia (GVL) effect] after allogeneic hematopoietic stem cell transplantation (HSCT). Alloreactive NK cells are considered rather save concerning the development of graft-versus-host disease (GVHD) (1–5), although a high number of activating killer-cell immunoglobulin-like receptors (KIR) (6) or extensive NK cell stimulation (7) might promote GVHD, maybe due to remaining T-cells in the graft. Shah et al. (7) found an association between infusion of activated NK cells and occurrence of acute GVHD (aGVHD): Children with ultrahigh-risk sarcoma received T-cell-repleted grafts from matched unrelated donors (URDs) or matched sibling donors with subsequent infusion of IL-15 and 4-1BBL preactivated NK cells. Five of nine patients developed aGVHD. Those effects were attributed to NK cell-mediated T-cell activation (7). The biology of NK cells is complex, but activation by human leukocyte antigen (HLA) *via* the group of KIR is considered to be a relevant mechanism of activation. Within this review, we will provide a summary of concepts of KIR-mediated NK cell activation and an overview of GVL effects in haploidentical (haplo), but especially in URD HSCT.

### Biology and Activation of NK Cells

Natural killer cells were named after their ability to kill infected or tumor cells without the need for prior antigen contact (8–10). They are defined by surface expression of CD56 and lack of CD3 (11). Unlike T cells, NK-cell receptors do not undergo rearrangement. In a process called licensing, NK cells with inhibitory receptors for present HLA class I (HLA-I) molecules (indicating “self”) are positively selected and stimulated for proliferation, leading to a licensed and self-tolerant subset. Missing inhibitory receptors against HLA-I do not lead to depletion but to a second subset of unlicensed but self-tolerant NK cells (12). Activation of NK cells might be initiated by antigen contact, but it is executed only after integration of abundant activating and inhibitory signals (13, 14). Today, several NK-cell receptors are known. Besides KIR, other NK-cell receptors that have been shown to have the potential to positively influence outcome after allogeneic HSCT are natural cytotoxicity receptors (15–17) as well as activating NKG2D (18) and DNAM-1 (19, 20) that bind to MICA/B and ULBPs or CD112/CD155, respectively. Both can be induced by DNA damage (21) and seem to play a role in negative regulation of T-cell responses (22) and acute myeloid leukemia (AML)/myelodysplastic syndrome immune evasion (15, 23).

### KIR and HLA

Killer-cell immunoglobulin-like receptors belong to type-I transmembrane proteins of the immunoglobulin-like receptor superfamily and recognize classical HLA-I molecules (14). The 15 KIR genes and 2 pseudogenes are located on chromosome 19q13.4. According to the number of extracellular immunoglobulin-like domains (D), the receptors are named KIR2D and KIR3D (24, 25). On the cytoplasmic side, they have either long (L) inhibitory or short (S) activating domains (14). Inhibitory KIR bind to the highly polymorphic regions of HLA-I molecules: HLA-A, B, and C (26), while the ligands for activating KIR are poorly defined (14, 27).

To facilitate description of KIR-ligands, HLA-C phenotypes can be grouped into HLA-C group 1 and 2 according to their respective KIR-binding motif. HLA-C group 1 contains all ligands with serine at residue 77 and asparagine at residue 80 of the α1 helix (HLA-C^asn80^), binding KIR2DL**2**/**3** and 2DS2. Members of this group are HLA-C*01/*03/*07/*08/*12/*14/*16. HLA-C group 2 (HLA-C^lys80^) has asparagine at residue 77 and lysine at residue 80 and contains HLA-C*02/*04/*05/*06/*15/*17/*18. They are ligands for KIR2DL1 and KIR2DS1 (28–31).

KIR3DL1 binds HLA-Bw4, and KIR3DL2 and 2DS2 bind HLA-A3 and A11 (14, 18, 32–38). Despite its structure, KIR2DL4 exhibits activating capacities and might bind soluble HLA-G (39–45). The KIR phenotype of an individual is his or her distinct set of inhibitory or activating KIR with an underlying distinct genotype (27, 46, 47). All genotypes can be summarized to a set of distinct haplotypes, which again result in the superordinated KIR haplotypes A or B (27, 46). KIR haplotype B is defined as the presence of KIR2DL5, 2DS1/2/3/5, or 3DS1, which have to be absent in KIR haplotype A (48). KIR2DS4 is the only activating KIR in haplotype A (46). KIR haplotype B/x (B/B or B/A) is found in about 30% of the Caucasian population (49). A more detailed analysis includes the information, whether the individual KIR is coded in the centromeric (Cen) or telomeric (Tel) gene motif of the KIR locus, resulting in Cen-A/A, Cen-B/x, and the respective Tel haplotypes (49–52). Thus, each individual expresses a certain KIR haplotype and a distinct HLA-C haplotype (C1/C1, C1/C2, or C2/C2). For prediction of alloreactive NK cell effects, the presence of HLA-C1, C2, and Bw4, as well as their respective KIR, are investigated (53). KIR2DL4 stimulation by HLA-G is considered to induce tolerance at the maternal–fetal barrier as well as IFN-gamma release of NK cells but not cytotoxicity (39, 43). KIR3DL2 and 2DS2 stimulation by HLA-A3 and A11 is also not in the primary focus of altering NK cell alloreactivity. KIR3DL2 has been identified as a surface marker in cutaneous T-cell lymphoma (54–56). For KIR2DS2, a reduced survival after URD-HSCT is suspected due to higher incidence of GVHD (57).

### Model Situations Predicting NK Cell Alloreactivity

**Figure 1:**
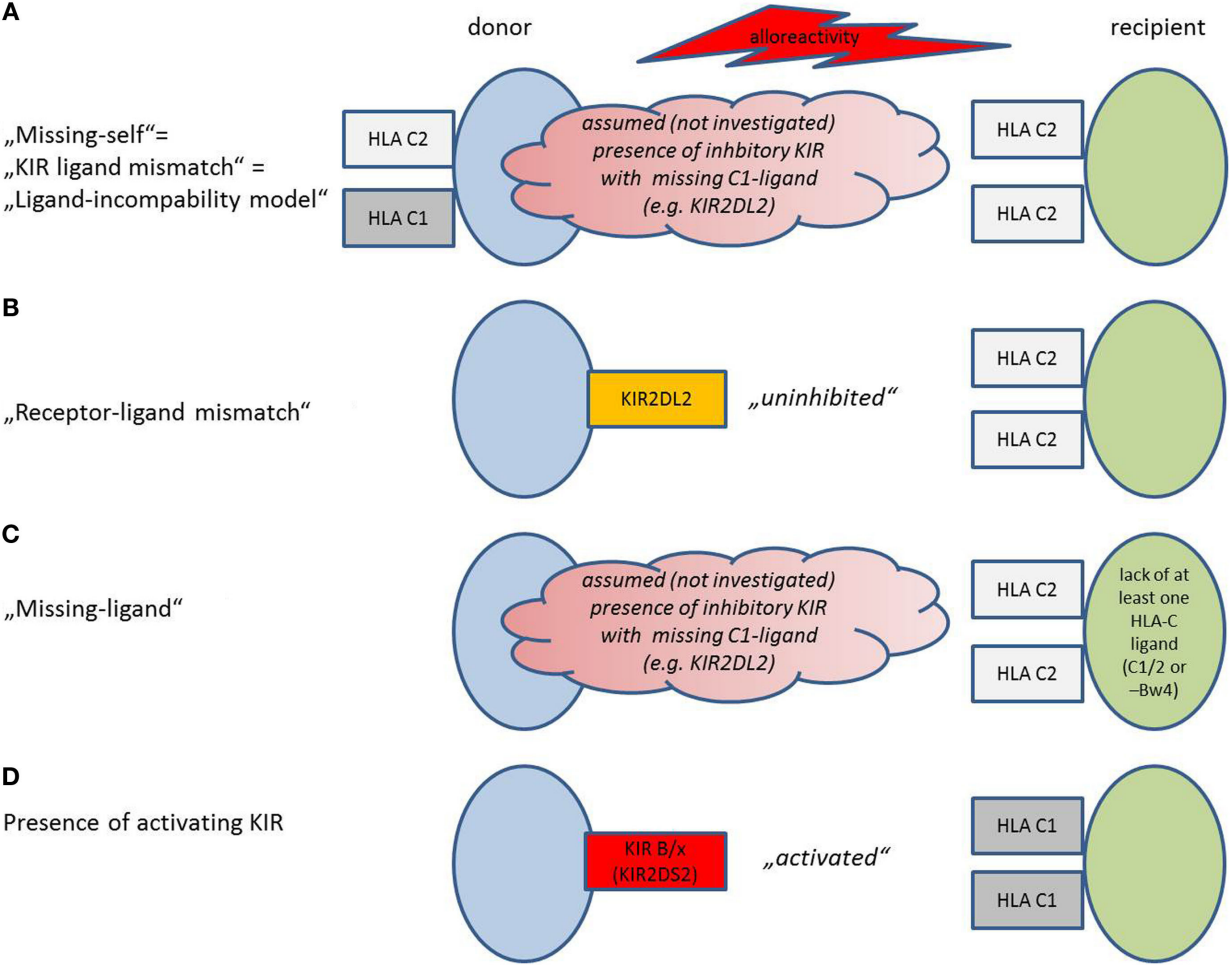
**Model situations that provoke natural killer (NK) cell alloreactivity**. Models are depicted as used in the present review, adopted and modified from Symons and Fuchs (53). Details concerning the activation mechanism are provided in the text. **(A)** Missing-self model, also described as “killer-cell immunoglobulin-like receptors (KIR)-ligand mismatch” or “ligand-incompability model”: Potential alloreactivity in the graft-versus-host direction is predicted by investigation of human leukocyte antigen (HLA) on donor and recipient. An HLA for inhibitory KIR that is present in the donor lacks in the recipient. The presence of the respective inhibitory KIR in the donor is assumed but not verified. **(B)** Receptor–ligand mismatch: NK cells become activated in the graft-versus-host direction, if they have an inhibitory KIR, for which the HLA ligand in the recipient is missing. Thus, the NK cells are “uninhibited.” Other than in **(A)**, KIR on donor cells and HLA on recipient cells are investigated, not “assumed.” **(C)** Missing ligand: If the presence of the respective inhibitory KIR is not evaluated, but assumed in a model where at least one HLA-ligand is missing (HLA-C1/2 or Bw4). Other than in **(A)**, only HLA on recipient cells but not on donor cells are evaluated. **(C)** The presence of activating KIR predicts alloreactivity in the presence of the respective activating ligand. KIR haplotype B/x contains more activating KIR than KIR haplotype A/A.

Different definitions of a mismatch between the donor’s NK cells and the recipient’s HLA exist, depending on the method that was chosen for KIR and HLA (HLA-C1, C2, and Bw4) evaluation (Figure 1).

#### Missing-Self/KIR-Ligand Mismatch (Figure 1A)

Alloreactivity was initially thought to be only dependent on lack of inhibitory HLA-I molecules in the recipient that are present in the donor (“missing-self” or “KIR-ligand mismatch” or “ligand-incompability model”) (53, 58–60). For evaluation of KIR-ligand mismatch, donor and recipient are screened for expression of HLA: NK cells from a HLA C1/C1 donor will be alloreactive against a C2/C2 recipient. If a recipient expresses HLA-C1, C2, and Bw4, he will be resistant toward NK cell killing, as seen in one-third of the population (61). It is assumed, but not verified that the respective KIR, necessary for alloreactivity, is present in the donor.

#### Receptor–Ligand Mismatch (Figure 1B)

The receptor–ligand model states that donor NK cells become activated in the graft-versus-host direction; if they have inhibitory KIR, for which the HLA ligands in the recipient are missing, the NK cells become “uninhibited” (4). Thus, in addition to the HLA status of the recipient, confirmatory KIR genotyping of the patient is required. Other than in the first model, KIR on donor cells and HLA on recipient cells are investigated, not “assumed.” This model can be considered as an improvement of the “missing ligand model.”

#### Missing Ligand Model (Figure 1C)

Here, only the recipients’ HLA are genotyped, and missing HLA-C1, C2, or Bw4 for inhibitory KIR predict an alloreactivity of the graft; the presence of the respective KIR that would bind to the missing HLA is only assumed (53).

#### Presence of Activating KIR (Figure 1D)

Some theories emphasize that to achieve NK cell alloreactivity, “un-inhibiton” of NK cells by missing inhibitory HLA ligands might not be sufficient. Activation requires additional stimulation of activating KIR in the graft (62). In this model, alloreactivity can be predicted by measurement of activating KIR on donor cells. Some studies increase the predictive validity by detection of the respective activating ligands on donor cells.

Therefore, we determine mismatches on the donor and recipient side between ligand–ligand, receptor–ligand, and receptor–receptor or identify activating KIR in the donor (49, 53). HLA and KIR can be investigated by genotyping, phenotyping, or functional NK cell assays to predict alloreactivity. We would suggest to follow the well-described methods of Ruggeri et al. for genotyping and phenotyping (61).

All approaches were initially tested in the haploidentical setting: The Perugia group suggests donor and recipient HLA typing to identify mismatch (2), followed by confirmatory donor KIR typing to verify a mismatch between donor KIR and recipient HLA ligand (“KIR-ligand mismatch” combined with a “receptor–ligand” concept) (61). They found ligand incompatibility between donor and recipient in haploidentical HSCT to be associated with increased GVL effects and lower relapse in acute leukemia (2, 4). KIR-ligand mismatch can be prevalent either in the graft-versus-host direction when the donor’s KIR ligand is not shared by the recipient or in the host-versus-graft direction when the recipient’s KIR ligand is not present in the donor. The St. Jude group rather focuses on receptor–ligand mismatch in the haplo setting (63), while the researchers from Minnesota implemented their strategy for the URD setting by selecting KIR B/x donors for HLA-C1-positive recipients for improved alloreactivity (50, 64, 65).

## Evidence of NK Cell-Mediated GVL Effects

### Lessons Learned from the Haploidentical HSCT Setting

Much knowledge concerning NK cell-mediated alloreactivity has been collected due to the implementation of haploidentical HSCT. To reduce the risk of GVHD, T-cell depletion was performed before graft infusion at the cost of graft rejection (66). These effects could be partially overcome by infusion of high numbers of stem cells (67). Ruggeri et al. were the first to show NK cell-mediated alloreactivity in the T-cell-depleted haploidentical graft (5). Facilitated engraftment as well as tumor lysis by NK cells occurred by donor grafts that were KIR-ligand incompatible in the graft-versus-host direction without occurrence of GVHD. Since then, many other groups have investigated the beneficial effect of alloreactive NK cells in the haploidentical HSCT (2, 68) and have refined criteria for potential donor choice (61). The results are promising for AML (4, 68, 69), while lymphoid malignancies have been shown to be resistant in some (2, 69) but not all cases (63, 70) for KIR-mediated NK cell effects. The present status of NK cell-mediated effects in haploidentical HSCT has been reviewed elsewhere (53, 71, 72).

### Results in the Unrelated-Donor HSCT Setting

After the identification of beneficial NK cell-mediated alloreactivity in haploidentical HSCT, efforts were made to adopt the findings for transplantations with URD (Table 1). Even though many patients already have the opportunity to receive a graft from an HLA-matched donor, donor choice by KIR repertoire is useful. Since HLA and KIR are inherited separately, approximately 75% of HLA-identical sibling donors and almost 100% of matched URDs will show KIR disparities and might therefore be a potential source for alloreactive NK cells (73, 74).

**Table 1:** **Studies on NK cell alloreactivity for unrelated donors**.

Reference	*N*	Median age (years)^a^	Disease (*n*)	Tx (*n*)	Model	Conditioning and graft source	Immunosuppression	Main results
Davies et al. (75)	175	17	CML, AML, ALL, MDS, others	MMUD (175)	KIR-L MM^b^	Myeloablative BM	TCD or CSA ± MTX	*Adverse KIR-L MM in myeloid malignancies*: Lower OS at 1 and 5 years (*P* < 0.01). No difference between KIR-L M/MM in any endpoint for total cohort

Schaffer et al. (76)	104	29	Diverse	MUD (62)/MMUD (42)	KIR-L MM	Myeloablative BM (80) PB (24)	MTX + CSA, ATG	*Adverse KIR-L MM*: Reduced OS and RFS

Giebel et al. (85)	130	18–20.5	Diverse	MUD (61)/MMUD (49)	KIR-L MM	Myeloablative BM (125) PB (5)	CSA, MTX, ATG	*Beneficial KIR-L MM*: Higher OS and RFS (*P* = 0.0007; 4.5 years). No influence of HLA-MM in the patients without KIR-L MM

Bornhauser et al. (77)	118	42–44	AML, CML, MDS	MUD (54)/MMUD (64)	KIR-L MM	Myeloablative BM (54) PB (64)	ATG (118)	*Adverse KIR-L MM*: Higher relapse for KIR-L MM (*P* = 0.02), but no difference in survival after KIR-L MM, MUD, and MMUD transplantation

Schaffer et al. (78)	190	35–39	Diverse	MUD (94)/MMUD (96)	KIR-L MM	Myeloablative (168)RIC (22) BM (118) PB (72)	CSA based (179) or TCD (11) plus ATG (all)	*Adverse KIR-L MM*: Higher infections, leading to increased TRM and reduced OS (*P* = 0.01), but no increase of relapse or GVHD

Venstrom et al. (96)	1,277	40.5–41.7	AML	MUD (664)/MMUD (613)	Missing ligand	Myeloablative (1,069)RIC/NMA (189) BM (689) PB (588)	Diverse, no ATG	*Adverse absence of C1*: HLAC2/C2 recipients have higher relapse than HLAC1/x recipients (*P* = 0.05)
					Receptor–ligand KIR genes			*Beneficial KIR2DS1 from C1/x donor* associated with lower relapse compared to absence of KIR2DS1 (*P* = 0.003) and lower mortality (*P* = 0.04) w/o higher high-grade aGVHD or TRM
								*Beneficial KIR3DS1* associated with lower mortality (*P* = 0.01) by lower TRM and aGVHD
								No predictive effects in ALL patients (separate cohort)

De Santis et al. (80)	104	24	Diverse	MMUD (104)	KIR-L MM	Myeloablative BM (65) PB (39)	No ATG	*Adverse: KIR-L MM* (HVG): Increased graft rejection
							BM: CSA, MTX (59), T-cell depletion (9)	Adverse KIR-L MM (GVH): Increased aGVHD grade 3–4
							PB: No CSA (39)	Adverse KIR-L MM (GVH or HVG): Increased TRM, decrease RFS
								*Beneficial high number of donor KIR*: Lower GVHD and improved survival

Giebel et al. (57)	111	18.5–21	Diverse	MUD (90)/MMUD (21)	Missing ligand	Myloablative BM (96) PB (15)	CSA, MTX, ATG	*Adverse absence of C1*: C2/C patients have lower OS and DFS, due to higher relapse

Sun et al. (97)	65	45–46	AML	MUD (39)/MMUD (26)	Receptor–receptor	Diverse	CSA + MTX (65)	*Prediction of incidence of aGVHD possible*: Activating KIR in the donor that lack in recipient and the lack of inhibitory KIR in the donor that are present in the recipient predict increased aGVHD
							No ATG or TCD	Indifferent results for KIR-L MM, missing ligand, number of activating KIR

Giebel et al. (98)	25	27	ALL, AML, MDS, CML, NHL	MUD (23)/MMUD (2)	KIR genes	Myeloablative BM (20) PB (7)	CSA, MTX, ATG	*Adverse presence of KIR2DS1*: Reduced OS and DFS due to increased GVHD and relapse
								*Indifferent presence of KIR2DS1*

Kröger et al. (79)	142	33	AML, MDS, CMML, CML, ALL	MUD (103)/MMUD (39)	KIR haplotype	Myeloablative BM (67) PB (75)	ATG, CSA, MTX	*Adverse KIR B/x*: Higher relapse than KIR A/A (*P* = 0.03), but only in AML/MDS/CML/CMML, not ALL, resulting in lower OS
					KIR-L MM			*Adverse KIR-L MM*: Higher TRM, lower OS, no increase of GVHD
								*Adverse KIR3DS1, 2DS1, 2DS5* in UVA, only 2DS5 in MVA, all resulting in higher relapse

Farag et al. (83)	1,571	59–68	AML, MDS, CML	MMUD KIR-L MM GVH (137)	KIR-L MM	Myeloablative BM	± T-cell depletion	*Indifferent KIR-L MM*: For KIR-L MM (GVH/HVG) as well as KIR-L M but HLA MM at HLA B ± C versus HLA- and KIR-L M grafts: Same rates of increased aGVHD grade 3–4, TRM, treatment failure, and overall mortality compared to HLA- and KIR-L matched grafts
				MMUD KIR-L MM HVG (170)				
				MMUD KIR-L M (260)				
				MUD (1,004)				

Hsu et al. (60)	1,770	34.5–35	AML, MDS, CML, ALL	MMUD (1,190)/MUD (580)	Missing ligand	Myeloablative BM or PB	T-cell replete grafts	*Beneficial: missing ligand in MMUD* (defined as homozygosity of recipient HLA-B or C epitopes) resulting in lower relapse (*P* = 0.004), but not for MUD
					KIR-L MM			*Absence of HLA-C2 or Bw4 associated with reduced relapse*, no survival benefit
								*Indifferent KIR-L MM* model in subgroup of 428 patients: no difference in relapse (but also not with applied missing-ligand model in same subgroup *P* = 0.07)

Miller et al. (93)	2,062	–	AML, CML, MDS	MMUD/MUD	Missing ligand	-	± ATG or TCD	*Beneficial absence of one ligand in early stage AML or MDS*: reduced relapse, independent from HLA match (C1/C2/Bw4)
								*Adverse absence of ≥1 ligand in CML*: Increased late-onset high-grade acute GVHD

Willemze et al. (82)	218	12.8–15	AML, ALL	MUD (42)/MMUD (176)	KIR-L MM	RIC (202)Myeloablative (6) CB (single)	CSA based (174) Other (44)± ATG (196)	*Beneficial KIR-L MM*: Improved DFS, OS, and decrease relapse

Gagne et al. (84)	264	24.5	Diverse	MUD (164)/MMUD (100)	KIR-L MM Missing ligand Receptor–ligand Receptor–receptor	Myeloablative BM	Unmanipulated BM	*Indifferent KIR-L MM* *Adverse missing-ligand*: Decreased survival but only in C1-deficient recipients, in myeloid malignancies *Adverse receptor–ligand mismatch: KIR3DL1* as well as KIR3DL1/3DS1 mismatch (GVH: D+ R–, absence of recipient HLA-Bw4) from a HLA-Bw4-negative donor is correlated with low OS in HLA-identical and high relapse in MMUD HSCT

Ludajic et al. (94)	124	42	Diverse	MUD	Missing ligand	Myeloablative (90)RIC (34) BM (54) PB (70)	CSA-based (124) ± ATG (30)	*Adverse absence of HLA-C2* in recipients of KIR2DL1-positive grafts or KIR A/A grafts: Increased aGVHD
								*Beneficial absence of HLA-C2* in recipients of KIR2DS2-positive grafts: Decreased aGVHD

Cooley et al. (64)	448	33–34	AML	MUD (209)/MMUD (239)	KIR haplotype KIR-L MM	Myeloablative BM (397) PB (51)	T-cell replete MMUD grafts	*Beneficial KIR B/x in KIR-L M HSCT*: Compared to KIR A/A higher RFS in KIR-L M (MUD and MMUD) but not in KIR-L MM (MMUD)
								*Beneficial survival rates for KIR2DL2 and 2DS2 positive grafts*

Cooley et al. (50)	1,409	19/39	ALL, AML	MUD (687) MMUD (722)	KIR haplotype	Myeloablative BM (942) PB (467)	T-cell replete MMUD grafts	*Beneficial KIR B/x*: Higher RFS in AML but not ALL
								Cen-B motifs improve outcome without increased aGVHD/cGVHD or TRM

Venstrom et al. (99)	1,087	35.3–37.5	AML, MDS, CML, ALL	MUD (670)/MMUD (417)	KIR genes KIR haplotype	Myloablative BM (1,050) PB (37)	CSA (751)	*Beneficial presence of KIR3DS1*: Same rate of relapse but reduced TRM and aGVHD, resulting in lower mortality in AML and MDS. Beneficial effects increase with copy numbers of donor KIR3DS1
							No CSA (120)	*Beneficial effect of KIR B/x (including KIR3DS1) similar but weaker*
							TCD (216)	

Kröger et al. (100)	118	51	MM	Unrelated (81)	KIR haplotype	Myeloablative (12)RIC (106) BM (13) PB (105)	ATG (110)	*Beneficial KIR B/x B in MUD*: MUD but not MMUD haplotype B/x reaches lower 1-year relapse than haplotype AA (*P* = 0.005), resulting in higher 5-year DFS (*P* = 0.009).
				Related (37)				

Venstrom et al. (96)	1,277	40.5–41.7	AML	MUD (664)/MMUD (613)	Missing ligand Receptor–ligand KIR genes	Myloablative (1,069) BM (689) PB (588)	CSA (346)	*Adverse absence of C1 and beneficial KIRSDS1*: Reduced risk of relapse, if the allograft was derived from an HLA-C1/x donor
							Tac (428)	*Beneficial presence of KIR3DS1*: Not lower relapse but reduced TRM and aGVHD, resulting in lower mortality in AML
							TCD (348)	

Cooley et al. (65)	1,532	Adults and children	AML	MUD (856)/MMUD (676)	KIR haplotype	Myeloablative	T-cell replete MMUD grafts	*Beneficial KIR B/x, adverse absence of C1*: Relapse protection improved by high KIR-B content in recipients HLA-C1/x but not C2/C2 (significant only in MMUD, not MUD). No effect of donor HLA
					KIR gene content			
					Missing-ligand			

Sobecks et al. (95)	909	56–57	AML, MDS	MUD (712)/MMUD (197)	Missing ligand	RIC BM (169) PB (740)	Diverse ± ATG (317)	*Adverse KIR2DS1 educated in a C2/C2 donor*: Higher GVHD and TRM without reduced relapse (AML)
								*Adverse ≥1 missing ligand or absence of HLA-C2*: Higher aGVHD (AML)
								*Indifferent* KIR centromeric gene content or donor activating KIR

Faridi et al. (49)	281	50	AML, ALL	MSD (153)/MUD (128)	Comparison of different models	Myeloablative BM (10) PB (271)	ATG, CSA, MTX	*Adverse KIR-KIR mismatch*: Increased cGVHD in HLA C1/x recipients
								*Beneficial ≥1 missing ligand*: Reduced relapse without improved OS
								*Indifferent* results for KIR B

Bachanova et al. (101)	614	48–52	NHL	MUD (396)/MMUD (218)	KIR haplotype	Myeloablative (253)RIC (361) BM (227) PB (387)	Diverse	*Beneficial KIR B/x in MUD HSCT*: Lower relapse after 5 years compared to KIR A/A donors (*P* = 0.5) with improved progression-free survival (*P* = 0.007)

Rocha et al. (81)	461	Adults and children	AML	MMUD (461)	KIR-L MM	Myeloablative CB (single)	With (145) or w/o (35) *in vivo* T-cell depletion in 3–5/8 MM cohort	*Adverse: KIR-L MM (HVG)*: At 3–5/8 HLA-MM level: Higher mortality (*P* = 0.008) and NRM (*P* = 0.008), no difference in relapse or GVHD in KIR-L MM versus KIR-L M (HVG) for AML and ALL
				3–5/8 HLA-MM (212)				*Indifferent KIR-L MM (GVH)*: No differences in GVH direction or higher HLA-matched subgroup or complete patient cohort

*ALL, acute lymphoid leukemia; AML, acute myeloid leukemia; ATG, antithymocyte globulin (rabbit ATG, Campath or OTK3); BM, bone marrow; Cen, centromeric; CML, chronic myeloid leukemia; CMML, chronic myelomonocytic leukemia; CSA, cyclosporine A; GVH, graft-versus-host direction; GVHD, graft-versus-host disease (a, acute; c, chronic); HD, Hodgkin’s disease; HLA, human leukocyte antigen; HVG, host-versus-graft direction; KIR, killer-cell immunoglobulin-like receptors; KIR-L M, KIR-ligand match; KIR-L MM, KIR-ligand mismatch; M, matched; MDS, myelodysplastic syndrome; MM, multiple myeloma or mismatch; MSD, matched sibling donor; MMUD, mismatched unrelated donor, defined as HLA 10/10 – 8/8 or not further specified; MTX, methotrexate; MUD, matched unrelated donor; MVA, multivariate analysis; NMA, non-myeloablative; n.s., not significant; Tel, telomeric; PB, peripheral blood stem cells; RIC, reduced intensity conditioning; TCD, T-cell depletion; Tx, transplantation details; UVA, univariate analysis*.

*^a^Median age was not always declared for the total cohort but only the investigated subgroups. In this case, age-age does not describe a range but the youngest and oldest median age stated*.

*^b^KIR-L MM in the GVH direction unless stated otherwise*.

### KIR-Ligand Mismatch Seems to Induce Adverse Effects in URD HSCT

Davies et al. (75) were the first to perform a retrospective analysis of patients with HLA mismatched URD HSCT, comparing KIR-ligand mismatch. In the analysis, no difference in any of the primary endpoints was achieved. Concerning the subgroup of myeloid malignancies, KIR-ligand mismatch resulted in worse OS at 5 years [13 versus 38%, *P* < 0.01, no use of antithymocyte globulin (ATG)], which was even more surprising. Others confirmed worse outcome for KIR-ligand mismatch in URD HSCT after conditioning with ATG (76–79) or without ATG (80), accompanied with higher infections in the early posttransplant period (78) or increased graft rejection, TRM, and GVHD (80). A recent study confirmed higher mortality and higher TRM without difference in relapse in 3–5/8 HLA-mismatched KIR-ligand mismatched (in the host-versus-graft direction) unrelated cord blood transplantations for AML and acute lymphoid leukemia (ALL) compared to KIR-matched cord blood, while no difference was found for mismatch in the graft-versus-host direction or in a higher HLA-matched subgroup or the complete patient cohort (81). The authors did suggest to not using KIR-ligand mismatch as a criterion for cord blood selection. An earlier Eurocord study (82) detected favorable outcome for KIR-ligand mismatched transplantations in AML and ALL but used lower HLA-resolution techniques.

No difference in mortality after either KIR-ligand mismatched or HLA-mismatched but KIR-ligand matched donor–recipient pairs was detected by a comprehensive study of CIBMT, EBMT, and the Dutch transplant registry (83), investigating the results of 1,571 patients with myeloid malignancies with or without T-cell depletion. KIR-ligand mismatch was associated with significantly higher high-grade aGVHD, just as HLA mismatch at HLA-C and/or B. No predictive effects of KIR-ligand mismatch on outcome after T-cell-repleted unrelated HSCT were detected in a retrospective multicenter study in France (84). Here, different models of NK cell alloreactivity were compared in a very heterogeneous cohort of patients. These investigations were partially designed as a response to the positive results in haploidentical HSCT and in a previous study by Giebel et al. (85) with different results: KIR-ligand mismatch in patients with myeloid malignancies achieved significant higher OS and RFS as well as lower TRM and relapse compared to HLA mismatch with KIR ligand match or compared to matched URD HSCT with the use of pretransplant ATG. The differing results could be only partially attributed to the use or sparing of ATG (85): Although toxic (86) or immunosuppressive (87, 88) on NK cells, ATG has been shown to accelerate NK-cell and B-cell reconstitution in some (89) but not all investigations (90, 91). It has also been shown to decelerate the recovery of CD4+ and CD8+ T cells (89, 91) while sparing effector-memory T cells and T-regulatory cells (91). The results indicated that knowledge from haploidentical cannot be transferred to unrelated HSCT without further adaptations (75). Grafts for haploidentical HSCT were mainly highly T-cell depleted and performed with high stem-cell doses as well as no or low immunosuppression, resulting in fast NK cell but slow T-cell reconstitution with low T-cell numbers and eradication of antigen-presenting cells by alloreactive NK clones (2, 67, 92). Therefore, the immunological environment during engraftment in haploidentical HSCT is much different from URD-HSCT.

### Missing-Ligand Model and Presence of Activating KIR Are Predictive for Outcome

Later, Hsu et al. (60) identified not only KIR-ligand mismatch but also missing KIR ligands as protective against relapse in HLA mismatched but not in matched URD HSCT. These effects were seen in myeloid and lymphoid malignancies and supported by later investigations by other authors (93). In the study by Hsu et al. (60), the absence of HLA-C2 or HLA-Bw4 KIR ligands was associated with lower relapse. Other authors confirmed the impact of HLA-C2: Absence of HLA-C2 in recipients of KIR2DL1-positive grafts resulted in higher incidence of aGVHD after myeloablative (94) as well as reduced intensity (95) conditioning. The absence of C1 epitopes, as seen for C2/C2 recipients, has been claimed responsible for poorer outcome (57, 65, 96). In search for favorable KIR in URD HSCT, Sun et al. (97) prospectively analyzed outcome of URD AML patients without *in vivo* T-cell depletion by ATG. According to the presence or absence of activating or inhibitory KIR in donor and recipient, they calculated a new predictive algorithm for GVHD, in which an inhibitory KIR in the donor that lacks in the patients has a negative value *vice versa* a positive value. On the other hand, they could not find other models such as KIR-ligand, missing-ligand, or high numbers of activating KIR to be predictive for aGVHD (97). In general, among the activating receptors, the presence of KIR2DS2 has been shown to be associated with lower OS and DFS as well as higher incidence of GVHD, resulting in high TRM (98). The alloreactivity of KIR2DS1 educated in a C2/C2 donor results in higher GVHD and TRM without reduced relapse (95). KIRSDS1 has been claimed responsible for reduced risk of relapse, if the allograft was derived from an HLA-C1/x donor (96), but did not show any beneficial effects in other investigations (98). The presence of KIR3DS1 was not associated with lower relapse but reduced TRM and aGVHD, resulting in lower mortality in AML patients (96, 99). KIR3DL1 and KIR3DL1/3DS1 mismatch in the GVH direction (donor positive, recipient negative, absence of recipient HLA-Bw4) from a HLA-Bw4-negative donor is correlated with low OS in HLA-identical and high relapse in HLA-mismatched URD HSCT (84). There are several other investigations apart from the environment of URD HSCT, which might be even more conflicting and difficult to transfer. Our early investigations showed the low-alloreactive KIR haplotype A to be associated with lower relapse after HSCT for leukemia (79), while in a later analysis, KIR haplotype B was associated with improved PFS and OS in patients with multiple myeloma (100). Cooley et al. (50, 64, 65) systematically investigated the influence of the KIR haplotype B. In summary, a high number of KIR haplotype B defining receptors, especially of those coded in the centromeric regions, showed beneficial effects on survival of HLA C1/x AML recipients after ATG-free HSCT without increased GVHD and without benefit of KIR-ligand mismatch. No positive influence of haplotype B was seen in recent investigations for leukemia (49) but in HLA-matched URD-HSCT of non-Hogdkin lymphoma patients, where KIR B/x grafts led to significant lower relapse after 5 years compared to KIR A/A donors (*P* = 0.5) (101). The role of KIR genotypes in matched unrelated and sibling HSCT has recently also been investigated by Faridi et al. (49). Their aim was to compare the predictive value of KIR-ligand mismatch (61) versus the “missing-ligand” hypothesis (63) or the advantage of a specific KIR haplotype (50, 64, 65). They found KIR–KIR match to be associated with lower cGVHD for HLA C1/x recipients as well as lower RFS. One or more missing ligand in the unrelated recipient for donor KIR resulted in reduced relapse (21.6 versus 63.6%, *P* = 0.001) and higher RFS without improved OS. None of the tested hypotheses had influence on OS, and no effect of donor KIR haplotype was detected.

## Future Directions

During the past years, improvement in understanding NK cell alloreactivity has been made by wisely modeled analyzes (49, 60, 84). Despite clinical relevance (102–105), we still know too little about the NK cell education after HSCT (95). The interplay of NK cells and T-cells after HSCT is still subject of further investigation (105), and as we now know about KIR expression on T-cells (106), we need to be precise in our technical methods. To overcome the problem of heterogeneity, we would suggest beginning with a simple multicenter prospective trial in adult patients with AML in first molecular complete remission, testing the hypothesis that the number of activating KIR in the unmanipulated graft improves overall survival without increasing GVHD. KIR and HLA of donor and recipient should be measured by high-resolution genotyping and phenotyping. Every patient should receive the same conditioning and first-line immune suppression.

## Conclusion

Due to heterogeneity of the conducted studies, a general recommendation cannot be made. In matched URD-HSCT, a donor with high numbers of activating KIR can be chosen to optimize patient’s chances for survival.

## Author Contributions

SH and NK contributed equally to the manuscript writing.

## Conflict of Interest Statement

The authors declare that the research was conducted in the absence of any commercial or financial relationships that could be construed as a potential conflict of interest.
